# A Review of Indocyanine Green Fluorescent Imaging in Surgery

**DOI:** 10.1155/2012/940585

**Published:** 2012-04-22

**Authors:** Jarmo T. Alander, Ilkka Kaartinen, Aki Laakso, Tommi Pätilä, Thomas Spillmann, Valery V. Tuchin, Maarit Venermo, Petri Välisuo

**Affiliations:** ^1^Department of Electrical Engineering and Energy Technology, University of Vaasa, Vaasa, Finland; ^2^Department of Hand Surgery, Tampere University Hospital, 33680 Tampere, Finland; ^3^Department of Neurosurgery, Helsinki University Central Hospital (HUCH), Helsinki, Finland; ^4^Department of Cardiosurgery, Helsinki University Central Hospital, Helsinki, Finland; ^5^Department of Equine and Small Animal Medicine, University of Helsinki, Helsinki, Finland; ^6^Saratov State University, Saratov 410012, Russia; ^7^Institute of Precise Mechanics and Control, Russian Academy of Sciences, Saratov 410028, Russia; ^8^University of Oulu, Oulu, Finland; ^9^Clinic of Angiosurgery, Helsinki University Central Hospital, Helsinki, Finland

## Abstract

The purpose of this paper is to give an overview of the recent surgical intraoperational applications of indocyanine green fluorescence imaging methods, the basics of the technology, and instrumentation used. Well over 200 papers describing this technique in clinical setting are reviewed. In addition to the surgical applications, other recent medical applications of ICG are briefly examined.

## 1. Introduction

 Fluorescence Imaging (FI) is one of the most popular imaging modes in biomedical sciences for the visualisation of cells and tissues both *in vitro* and *in vivo* [1]. The benefits of FI include

- high contrast, that is, signal to noise ratio (SNR): only the target, not background, is visible because separate wavelengths are used for illumination and recording (cf.  Figure 4);
- high sensitivity: extremely small concentrations can often be made visible;
- Gives molecular information: makes some (bio) chemistry spatially and temporally visible;
- great tools for research: several possible imaging modes, most of which are unique;
- cheap: the optical instrumentation and computing needed are quite simple;
- easy to use: resembles classical staining.

Fluorescent imaging is a relatively recent imaging method and thus still developing in many ways. This is especially true for indocyanine green (ICG) imaging in its new clinical applications recently proposed in various branches of surgical medicine, although it has been used in some clinical applications routinely already for almost sixty years. Thus, ICG is well known in its established clinical applications, which greatly facilitates its introduction to new applications. From an engineering point of view, image and video processing seems to be among the main areas in which ICG imaging (ICGI) has potential for major developments, for example, for analysis of ICG fluorescence dynamics [2] (cf.  Figure 2). This means, among other things, that a lot of computing development work is still needed for a broader acceptance of various emerging ICG-based medical imaging methods [3]. 

### 1.1. Indocyanine Green Angiography

 Indocyanine green has been used for decades in ophthalmology for imaging retinal blood vessels, that is, in retinal angiography. However, fluorescein operating in visual wavelengths has been much more popular in retinal angiography partly because it is visible without any electronic cameras. However, the objects of imaging, retinal layers, with fluorescein and ICG somewhat differ. ICG gives information about deeper lying blood veins because it operates in near infrared (NIR), in which tissues are much more translucent than in visual wavelengths.

**Figure 1:**
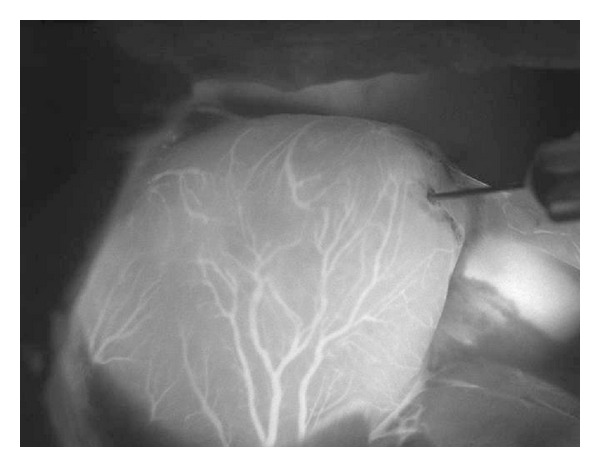
A typical ICGA image: heart of a rat. Coronary arteries clearly visible. Liver shining on the right. Magnification 20×. Image taken by Dr. Outi Villet at HUCH by our prototype microscope device shown in Figure 6.

**Figure 2:**
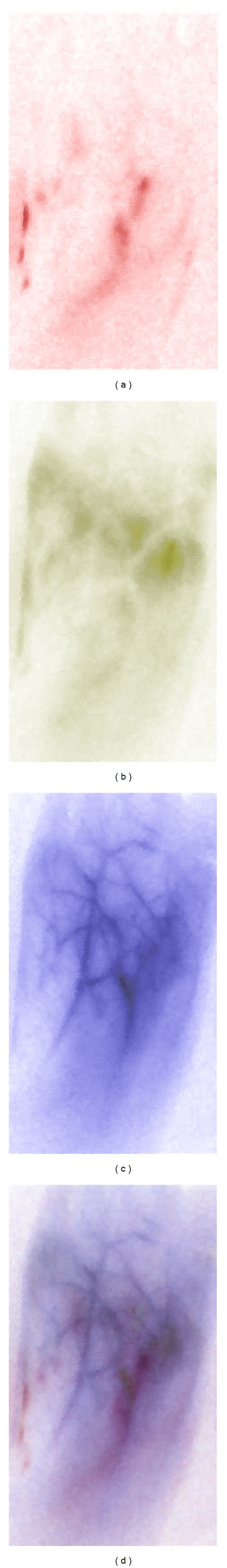
Simple image processing and pseudocoloring: ICG-VA frames of a leg (toes up) after the injection of ICG: (a) at about 30 s showing deep lying arteries in red, (b) at about 60 s showing mainly capillaries in yellowish green, (c) at about 90 s showing mainly subcutaneous veins in blue, and (d) fusion of the first three images. Image processing steps: negative of the original image and some intensity remapping. Fusion by using CMYC model. For more information see [5]. The original B/W images were taken with PDE by Dr. Hiroaki Terasaki (visiting HUCH from Tokyo Medical and Dental University Hospital of Medicine) and later processed by one of the authors (P. Välisuo).

The principle of fluorescence imaging used in ICG angiography (ICGA) is simple: illuminate the tissue of interest with light at the excitation wavelength (about 750 to 800 nm) while observing it at longer emission wavelengths (over 800 nm; Figure 4). To create a simple ICGA device, only a couple of filters are needed in addition to a proper camera and a light source, which can be quite small and suitable even for portable use [4]. The filters are needed to prevent the mixing of the excitation (strong) and fluorescing (weak) rays to sum at the sensor. Even if the fluorescence is only a small fraction of the excitation intensity (Table 9: row 1 versus row 10), a surprisingly good signal to noise ratio (SNR) is attained: a brightly fluorescing object, mostly blood vessels containing ICG, can be clearly seen on an almost black background (see Figure 1). Without the filters, the weak fluorescence image cannot be seen among the strong reflection of the excitation light. 

Indocyanine green dye was developed for near-infrared (NIR) photography by the Kodak Research Laboratories in 1955 and was approved for clinical use already in 1956 [6, 7]. However, it took over ten years before ICG was used for angiography [8]. For retinal angiography it has been used from early 70 s [9].

### 1.2. Related Work

 A few reviews of ICG and ICGA have been published. Those are briefly reviewed in what follows. Frangioni gives a review on *in vivo* fluorescent imaging including ICG-assisted imaging [10]. Amiot et al. give a review of the different NIR fluorescent materials developed and proposed for biomedical imaging [11]. Choyke et al. give a review of the toxicity of organic fluorophores including ICG used in molecular imaging [12]. For a recent review of ICG in retinal angiography, see for example, [13–15]. ICG, and infracyanine green in macular hole surgery are reviewed in [16]. ICG and some similar dyes in vitreoretinal surgery are reviewed in [17]. A short overview of early works on fluorescence-enhanced contrast imaging and tomography is given in [18]. A recent review of ICG in assessment of liver function is given in [19], and a personal history view on ICG in liver monitoring is given by Paumgartner [20]. Houston gives an overview of *in vivo* small animal studies on fluorescent contrast agents [21]. te Velde et al. have recently briefly reviewed all papers regarding fluorescent dyes in surgical oncology [22], Schaafsma et al. have reviewed ICG in oncologic surgery [23], Polom et al. ICG usage in oncology and especially in sentinel lymph node biopsy (SLNB) [24] and Luo et al. NIR dyes including ICG in cancer targeting and imaging [25]. In a recent review Kaiser et al. review optical methods, including ICG imaging, in noninvasive assessment of burn wound severity [26]. An excellent and illustrative review of ICG in clinical imaging of the lymphatic system is given recently by Marshall et al. [27]. National Library of Medicine maintains a database of contrast agents called MICAD [28]. 

### 1.3. ICG Publications

 To get an idea of the volume of ICG-related research activities, the number of ICG-related publications in several databases (PubMed, ISI, IEEE, and SPIE) was collected in Table 1 and classified according to the main application areas. As anticipated, most research on ICG seems to be related to clinical sciences and not to, for example, engineering, optics, spectroscopy, or imaging, which indicates that there is still much work left to reveal all the technical potential of ICG. For instance, most of the works on image processing deal with ICGA of the retina only. On the other hand, the long and routine use of ICG in some clinical applications, such as retinal imaging, has provided us with much invaluable knowledge and experience useful in the development of new clinical applications, which are anticipated to be introduced exceptionally swiftly and at the same time at both relatively low risk and cost.

The number of annual ICG publications according to PubMed is given in Table 2. The increase of publications has been about 10 papers per year. This number is expected to increase due to the emerging clinical applications described later in this paper. We can already (Fall 2011) see a considerable increase of papers for the years 2009 and 2010. According to Espacenet (6.5.2010), there are over 170 ICG-related patents.

## 2. Properties of Indocyanine Green

 The principal advantages causing the rapid acceptance of ICG were the presence of the absorption maximum, around 800 nm, the confinement to the vascular compartment through binding with plasma proteins, the low toxicity (LD_50_ of 50–80 mg/kg for animals http://www.drugs.com/pro/indocyanine-green.html), and the rapid excretion, almost exclusively into the bile.

ICG fluoresces at about 800 nm and longer wavelengths. The exact shape of the spectra depends somewhat on the chemical environment and physical condition of ICG molecules like temperature and ICG concentration. The spectra are also smoothly varying, thus the exact wavelength values given in the literature somewhat vary depending also on the excitation light spectra and the filters used.  Table 5 gives some excitation and observation wavelengths used in different ICG imaging instruments. The sensitivity of fluorescence spectra on molecular environment means that ICG is a potential molecular probe [29]. This has not yet been used in clinical applications. This is obviously one potential direction of ICG imaging development. Related to this direction is the need for better understanding of the binding of ICG molecules in different cells and tissues. This is clearly an arena for some further systematic basic research using fluorescence microscopy that may later possibly even lead to some major imaging innovations in biomedical applications.

ICG has several clinically excellent properties, which has been thoroughly verified during its long clinical use:

- patient safety: nontoxic and nonionizing,
- ideal for angiography: binds efficiently to blood lipoproteins, that is, it does not leak from circulation,
- short life time in blood circulation allowing repeated applications,
- good SNR: there is not much NIR autofluorescence in tissue giving low noise background,
- deep imaging: operates in tissue optical window (NIR), and
- simple and cheap imaging devices (Hamamatsu: [30]).

What is so new in ICG angiography? Recently new successful medical applications, mainly in surgery, have been introduced. Some of the ICG's subexcellent properties provide further challenges to research and engineering development:

- ICG is very recent in many applications such as cancer treatment, reconstructive surgery, and even in cholecystectomy,
- ICG needs some NIR imaging device to be visible,
- for some applications ICG seems to need online illumination control facility,
- clinically usable chemical derivatives for more specific physicochemical imaging do not yet exist,
- ICG injection solution contains some sodium iodide; thus, an allergic reaction is possible,
- ICG is unstable in solutions (10 h) and when exposed to light, and
- ICG has nonlinear fluorescence quantum yield versus concentration.

The development work for creating even better NIR contrast agents is going on in a few laboratories. Some of the proposed new molecules are based on ICG, while there are also totally different approaches such as quantum dot-based contrast agents [31, 32].

### 2.1. Structure and Stability

 Indocyanine green is a tricarbocyanine dye having a molecular weight of 751.4 Da. It is a negatively charged ion that belongs to the large family of cyanine dyes [33]. Dry ICG is stable at room temperature. This is also the form of pharmaceutically available ICG. ICG is soluble in water (1 mg/mL) but is not readily soluble in saline. Therefore, ICG should first be dissolved in water and only after this diluted with saline if an isotonic solution is needed (Sigma-Aldrich). Some chemicals, such as sodium polyaspartate (PASP), can be used to stabilise ICG in water and blood solutions, for example, when blood samples should be stored for several days. The use of PASP has been demonstrated also *in vivo* for a rat model [34, 35]. The chemical decomposition of ICG can be inhibited by sodium azide NaN_3_, a quencher of singlet oxygen, that is, an antioxidant [36]. Also storage of the ICG solution at low temperature (4°C) inhibits decomposition, while storage at room temperature facilitates decomposition [7].

#### 2.1.1. Spectral Stability

 In aqueous solutions, ICG molecules tend to aggregate, which influences their optical properties [37]. The aggregation depends on concentration and time; thus, ICG solutions do not follow Lambert-Beer's law above 15 mg/L in plasma [38]. The spectral stabilisation is fastest when ICG is dissolved in distilled water, and thus Landsman et al. do not recommend adding isotonic saline and/or albumin to the injectate, when fast spectral stability is essential, for example, when using ICG for quantitative purposes [38]. In tissues and cells the NIR absorption peak, due to binding with cell proteins, is moved to longer wavelengths (810 nm) [39].

#### 2.1.2. Photochemical Stability

 When excitated ICG is supposed to produce singlet oxygen, which is a strongly cytotoxic agent. Engel et al. have recently studied the stability of ICG when exposed to light and the production or the consequences of singlet oxygen production of ICG [7]. According to their observations the decomposition of ICG is due to singlet oxygen, but it seems that the singlet oxygen is immediately bound to the decomposition products of ICG itself. Therefore, it seems that ICG is not a very good source of singlet oxygen. This has two main consequences with respect to clinical applications: firstly, ICG can be used without much worry of phototoxicity due to singlet oxygen production; secondly, when ICG is used as a photodynamic or photothermal agent, its decomposition products may be the main cause of phototoxicity. The decomposition products thermally decompose further to several carbonyl compounds. However, according to a recent study by Tokuda et al., ICG seems to be somewhat phototoxic for the retina [40].

Engel et al. tested several solvents for light-induced decomposition of ICG. What is again interesting and encouraging for angiography applications is that ICG in blood plasma was found to decompose so that only a small amount of decomposition products were recorded when compared to ICG in water. They suggest that the singlet oxygen produced is quenched by some plasma proteins thus inhibiting ICG decomposition by singlet oxygen [7]. Very recently Sato et al. studied the effect of broadband light on ICG toxicity by filtering the long wavelengths focused on cultured Müller cells. According to their observations filtering prevents phototoxicity [41].

#### 2.1.3. Protein Binding and Fluorescence Life-Time

 The important property of fast binding to plasma proteins, especially lipoproteins, [42–44] makes repeated intraoperational applications of ICG possible. The binding to plasma proteins does not seem to alter protein structures, which is one sign of nontoxicity [45]. It seems that ICG actually binds to the lipids of lipoprotein complexes (*β*-lipoprotein [43]), and that the bind results in more intense fluorescence than ICG bound to for example, free cholesterol [44]. Binding to blood proteins also shifts, slowly, taking several minutes, the absorption peak, at 780 nm, towards longer wavelengths, to 805 nm [46]. The absorption peak maximum was observed at 810 nm in the epidermal cell cultures [39], and at 805–810 nm in the human skin *in vivo *[47, 48]. The emission peak is also shifted similarly [14]. Not only the shape of the spectra is influenced by the chemical environment, but also the fluorescence life-time changes, a fact which can be used to probe the molecular environment of ICG and similar dyes [49].

### 2.2. Physiology and Pharmacokinetics

 ICG does not have any known metabolites, and it is fast extracted by the liver into bile juice. The transport is done by a protein called glutathione S-transferase without [7] modification. Caloric restriction seems to significantly increase the plasma clearance rate at low doses (0.5 mg/kg) [50]. The protein spectra of different liver diseases also affect ICG protein binding in blood [43, 51]. Reekers et al. provide a recent study of the plasma disappearance rate for ASA physical status I-II patients [52].

 The typical dye concentrations used for *in vivo* retinal and choroidal angiography are in the range of 20–25 mg/mL of ICG applied by injection into a peripheral arm vein [46]. For studies of hepatic function an intravenous injection dose is calculated on the basis of 0.5 mg/kg of body weight. In cardiac output and blood volume monitoring the total dose of dye injected should be kept below 2 mg/kg. No significant toxic effects have been observed in humans with the high dose of 5 mg/kg of body weight [53].  Table 3 gives a brief overview of toxicity studies done with ICG.

### 2.3. Penetration

 ICG works in the so-called tissue optical window, that is, the NIR light used both in excitation and fluorescence penetrates tissue several millimeters or even further. This translucency helps to observe, for example, vascular structures that might be buried in clots or dura [62, 63]. The penetration depth of light energy into skin and underlying tissues can be calculated on the basis of *in vivo* measurements of optical density *OD* (accounting scattering and absorption) of the human skin and underlying tissues defined as *OD* = log⁡_10_ *I*_0_/*I*_*t*_, where *I*_0_ is the measured back reflected intensity, and *I*_*t*_ is that of the reference. Such measurements done for 12 healthy young subjects at 775, 807, and 827 nm gave the following dependencies for a 3 mm layer of subcutaneous fat: *OD*_775_ = 3.2; *OD*_807_ = 2.4; *OD*_827_ = 1.6 [64]. The low absorption and high scattering allow for providing smooth and intensive enough indepth irradiation of skin tissue due to the photon recirculation effect [65].

### 2.4. ICG Derivatives and ICG-Like Contrast Agents

 While NIR fluorescence (NIRF) imaging has recognised potential, only ICG is a clinically approved NIRF dye. Perhaps in the future there will be a larger set of NIRF dyes. At least work on developing new NIRF dyes has been going on and has already introduced several potential NIRF dye candidates. Here we will only briefly review some recent development of ICG derivatives.

 While ICG is rapidly bounded with lipoproteins in blood, it is natural to combine ICG with nanoparticles of lipoproteins [66]. Lipid nanoparticles and micelles have been doped with ICG [67–70]. Ogawa et al. have conjugated ICG with several antibodies in order to target ICG to cancer cells. However, ICG conjugated to protein usually markedly looses its fluorescence. Therefore, in order to have high fluorescence efficiency, the ICG-antibody complex should be dissociated so that ICG can be used as an *in vivo* molecular imaging probe [71]. S. Achilefu's group has recently conjugated ICG with folate-polyethylene glycol for tumor targeting [72]. Ebert et al. have compared the pharmacokinetics of ICG to its hydrophilic derivative called SIDAG with a mice model for breast cancer imaging [73].

Several encapsulations have been implemented with ICG [74–78]. Makino et al. have labeled lactosome with ICG. The labeled lactosome was found to be stable in blood circulation and gradually accumulated specifically at a mouse model liver tumor site [79]. Barth et al. have engineered calcium phosphosilicate composite nanoparticles embedding ICG for targeting human breast and pancreatic cancers [80].

 Infracyanine green (IfCG) (Laboratoires SERB, Paris, France), also known as IFC green, is ICG without iodine. It is believed that IfCG is less cytotoxic in macular applications because 5% glucose solution instead of pure water is used as its solvent. According to [56], IfCG is less than ten times as retina cytotoxic as ICG. Infracyanine green was used in the study of macular pucker surgery [81]. The absorption and emission spectra of the commercial ICG and IfCG products in several solvents and concentrations are compared in [82].

## 3. Instrumentation

In this section, an overview of ICG imaging from the instrumentation engineering point of view will be given. Indocyanine green imaging belongs to the class of optical fluorescence imaging. Correspondingly, when used with an operational microscope it closely resembles fluorescence microscopy. Thus, the instrumentation needed is similar or even exactly the same as that for fluorescent imaging in general, or fluorescence microscopy in particular.

### 3.1. Fluorescence Imaging

 As a rule, fluorescence microscopy is done so that both visible or excitation and fluorescence images are displayed together as one image. The fluorescence image alone may contain only a few details so that the visible image greatly helps to locate the fluorescing parts with the help of the landmarks seen in the visible image. Typically the fluorescence channel is shown, rendered, in colors like vivid green, having a striking colour contrast to the visible image of tissues. This kind of visualisation is especially important in intraoperational use, where the fluorescing parts, like blood veins, should be recognised easily and immediately. In order to be able to combine the two images, they should be aligned correctly. This is called image registration, and it is generally a computationally hard image processing operation [83, 84], while the rendering of the two images for display is a straightforward operation.

**Figure 3:**
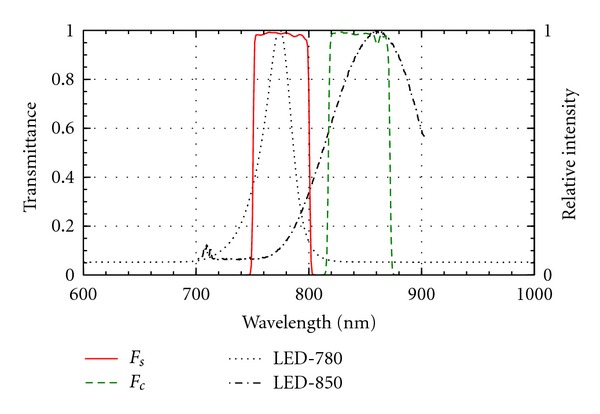
The transmission of the ICG filter pair (*F*_*s*_: high-pass filter for source; *F*_*c*_: low-pass filter (barrier) for camera) and the emission spectrum of two NIR LEDs having the nominal peak wavelengths of 780 and 850 nm and full width at half maximum (FWHM) bandwidths correspondingly of 30 and 95 nm.

However, the image registration problem can be totally avoided by optical means by using an ordinary beamsplitter, which is a dichroic mirror splitting and filtering the beam into two parts: one for the visible camera and the other for the NIR camera. This means that both cameras see exactly the same field of view (FOV), and no registration is needed, provided that the cameras have identical optics and are located correctly with respect to each other. In addition to the beamsplitter, suitable exchangeable filters embedded in the optics or in a separate filter cube, which is the usual arrangement in fluorescence microscopy, are used in front of the cameras to block unwanted wavelengths from entering the sensors [85]. The filter is especially important for the NIR camera, so that the excitation light does not mix with the fluorescence signal because both are summed at the sensor and inseparable in the resulting electronic image. Visible range cameras usually already contain filters that block most of the NIR radiation that would otherwise be summed to a varying extent to the different RGB channels of the visual image, thus distorting its perceived colours. The main difference between a traditional fluorescence microscope and an operational microscope doing ICG fluorescence imaging with a beam splitter is that the illumination, which is episcopic in the fluorescent microscope and thus done via the beamsplitter, is replaced by an ordinary colour camera, while the illumination can be done by an external light source [86]. The excitation light should not contain fluorescence wavelengths, they should originate only from the fluorescing ICG. Thus, a filter is needed to block longer wavelengths from the launched excitation light, when using a broad spectrum light source. Ideally the two filters should divide the spectrum into two nonoverlapping bands (Figure 3). This can be best done using interference filter pairs, which can be tailored for any wavelength range and which can have a very narrow transition band. Commercial interference filter pairs are also available for ICG fluorescence, separating the spectrum at about 800 nm (Chroma Technology, Brattleboro, VT, USA) (see Figure 3). When using a light source with a narrow spectrum, a laser, there is no need to use any excitation light filter. The use of a beamsplitter is a particularly simple and practical way of solving the otherwise challenging image registration problem and allows an illustrative blending of the colour image and the ICGA to be easily done online, which is often vital for critical intraoperational use (Table 4).

### 3.2. Example of an ICGA Device Design

**Figure 4:**
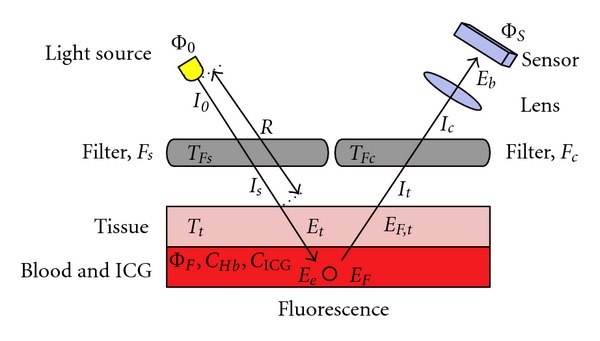
The principle of fluorescence imaging. The radiation from the light source is filtered by a high-pass filter, *F*_*s*_, to remove the fluorescent wavelengths. The blood and ICG suspension under a tissue absorbs the excitation wavelengths and emits in fluorescent band. The emitted light is received by the sensor through a low-pass filter, *F*_*c*_, to remove the excitation light reflected from the source.

**Figure 5:**
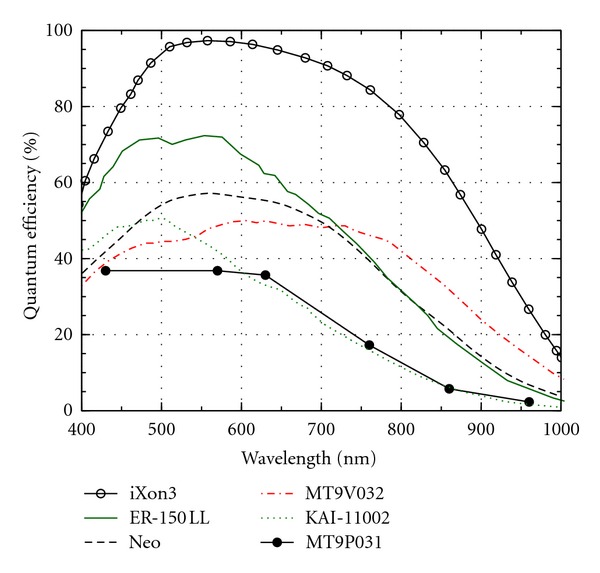
The quantum efficiencies of different sensor technologies in VIS-NIR range. iXon3 is an electron multiplier CCD, ER-150 LL is Hamamatsu biomedical CCD sensor, Neo is a scientific CMOS sensor, MT9V032 is a CMOS sensor for surveillance, KAI-11002 is a standard CCD sensor, and MT9P031 is a standard consumer CMOS sensor.

 In this section, we will, in principle, design a simple ICGA device. The principle of fluorescence imaging is given in Figure 4. In order to see the fluorescence, which has only a fraction of the intensity of the excitation light, the latter should not contain any fluorescence wavelengths. If a broadband light source, like a halogen lamp, is used, there should be a filter to cut the longer wavelengths (*F*_*s*_). In the case of using lasers, which are monochromatic, no filter is usually needed. On the camera side complementary filtering is always needed, that is, the excitation wavelengths, and possibly shorter wavelengths should be cut off (*F*_*c*_). As can be seen in Table 5, there is some freedom when selecting the exact filter wavelengths. In addition to the filter wavelengths we should also look at the wavelength dependence of the light sources, filters, and cameras. In an ideal case no excitation light is recorded by the camera while as much as possible of the fluorescence is recorded. This is obviously a two-goal optimisation problem: minimise excitation light leakage while maximising recorded fluorescence light. What makes this a nontrivial technical problem is the shape of the spectra of each component needed and the other properties of the components affecting recording. For example, the quantum efficiency of the silicon semiconductor-based image sensors in NIR band typically strongly decreases when the wavelength increases. The quantum efficiency means the fraction of photons striking the sensor that are actually recorded. For silicon sensors, it is in the visible wavelengths typically around 70%, while for NIR wavelengths around 700–900 nm it may range from 50% down to 10% or even less (Figure 5).

#### 3.2.1. Light Sources

Table 6 describes the basic properties of light sources available for fluorescence imaging. As we have seen in Table 5, all the basic light source types have been used in some of the existing ICGA implementations. Most frequently LEDs and halogen lamps have been used for illumination. In some experiments also lasers, mainly semiconductor diode lasers, resembling LEDs, have been used. In this example, we will look closer at LEDs. LED light is not totally monochromatic but contains wavelengths typically having a bell-shaped spectrum (Figure 3), which should not overlap too much with the camera filter spectrum (*F*_*c*_) (Figure 3).

If a visual image is recorded or observed, we naturally need a white light source. Note, that most microscope lights filter out NIR wavelengths at least partly.

Figure 3 shows the spectrum of an LED having a nominal (peak) wavelength of 780 nm (LED 780-66-60, Roithner Lasertechnik GmbH, Vienna, Austria). The measurement was done with an HR4000 Spectrophotometer (Ocean Optics, Dunedin, FL, USA). As can be seen, the wavelength range is over one hundred nanometers with 30 nm bandwidth (FWHM).

#### 3.2.2. Cameras

 Every CCD or CMOS camera is, in principle, able to record NIR. However, most cameras are prevented from doing so by a filter that cuts NIR wavelengths, otherwise the superimposed NIR image would badly interfere with the visual image. The most important parameters of the camera sensors are resolution, signal to noise ratio (SNR), and quantum efficiency. The parameters affecting the SNR are the resolution of the ADC converter, read noise, dark current, and quantum well depth of the sensor. These parameters for some selected sensors are listed in Tables 7 and 8: MT9P031 and MT9V032 (Aptina Imaging, San Jose, CA, USA) are typical complementary metal oxide semiconductor (CMOS) sensors, except that the NIR response of the latter is enhanced. The machine vision camera, Elphel NC353L (Elphel Inc. West Valley City, Utah, USA) includes the MT9P031 sensor. The KAI-11002 (Kodak, New York, USA) is a typical Charge Coupled Device (CCD) sensor. iXon3 and Neo sensors (Andor Technology plc, Belfast, North Ireland) are meant for scientific imaging, where high sensitivity is needed. The iXon3 is based on Electron Multiplier CCD (EM-CCD) technology, whereas Neo is based on the scientific CMOS (sCMOS) sensor. The FL-280 and ER-150 are corresponding sCMOS and CCD sensors from Hamamatsu (Hamamatsu Photonics K.K, Shizuoka, Japan).

While practically all silicon-based cameras are somewhat sensitive to near-infrared, when they do not have a filter to block NIR wavelengths, unfortunately the quantum efficiency tends to decrease quite rapidly by increasing wavelength (Figure 5). This decrease of quantum efficiency is an essential issue when designing ICG imaging because the fluorescence peak is quite broad and extends far beyond 800 nm, where the efficiency is quite low. Therefore, the nominal wavelengths of the light source and filters should be as short as possible, which is in contradiction with the good separation of excitation and fluorescence and the fact that the absorption maximum of ICG should be close to the nominal wavelength of the excitation light source. The quantum efficiency of ICG is quite low, about 0.3% in water and 1.2% in blood [104]. This constrains camera sensitivity especially in video applications. Cooled CCDs are often used to increase the signal to noise ratio (cf.  Table 5). Sometimes image intensifiers (night vision) are used to increase sensitivity for video recording and to allow low doses of ICG (microdosing) [21, 27, 105, 106].

#### 3.2.3. Filter Design

 For the LED source of Figure 3 we need a filter that effectively blocks all wavelengths longer than 800 nm. It seems that the most suitable off-the-shelf filter can be found from Chroma (Chroma Technology, Brattleboro, VT, USA), specially tuned to ICG fluorescence. The filter HQ845/55 m, which is of the interference type, has quite a sharp pass band between 820 and 870 nm, and when comparing the spectra of the LED (LED: 780, 66, and 60) we are using and this filter we can see that their spectra somewhat overlap (Figure 3).

A rigorous approach to ICGA system design would include numerical analysis of the spectra of the light source, filters, and the camera in order to find the optimal nominal wavelength of the components. However, in this study we have simply resorted to those components that were easily available and which seemed to fit with each other well enough.

 Photometric formulas can be still used in developing practical rules of thumb to estimate the effect of different components of the ICG imaging system as follows.

 The radiant flux of illumination source (cf.  Figure 4), Φ_0_ [W], distributed over a solid emission angle *Ω*_0_ [sr], forms the radiant intensity of *I*_0_ = Φ_0_/*Ω*_0_ [W/sr] (Table 9; row 1). *I*_0_ travels through the excitation filter, *F*_*s*_, the transmittance of which is *T*_*F*_*s*__ [unitless], attenuating to *I*_*s*_ = *I*_0_ *T*_*F*_*s*__, (Table 9; row 2) and hits the tissue surface. The irradiance of the tissue, *E*_*t*_ [W/m^2^], depends on the distance, *R* [m], and angle of the incidence, *θ*, as follows: *E*_*t*_ = *I*_*s*_ *Ω*_0_cos⁡(*θ*)/*R* ^2^. The tissue attenuates the incident irradiance by the factor of *T*_*t*_. For 1 mm of aorta tissue, the *T*_*t*_ ≈ 0.45 [1/cm] [107] (Table 9; row 4). 

Part of the incident irradiance is absorbed by blood and ICG and part of it will be diffusely reflected due to the scattering of the red blood cells (RBCs). The intensities of the excitation and fluorescence fields can be calculated using diffusion theory [108–110]. The scattering coefficient of red blood cells and absorption coefficients of hemoglobin, needed in the diffusion model, are listed in [111, 112]. Correspondingly, the absorption properties of ICG are listed in [113]. According to diffusion theory, the diffuse reflectance, excluding the fluorescence, is *R*_*d*_ ≈ 0.15. When the fluorescence is included, the total reflected and emitted intensity *R*_*t*_ is slightly higher. The intensity of the fluorescence emission is *E*_*f*_ = (*R*_*t*_ − *R*_*d*_)*E*_*e*_ ≈ 0.0027*E*_*e*_. Increasing the ICG concentration increases *E*_*e*_ quite fast in low doses, for example, when *C*_ICG_ = 6.5  *μ*M/L (0.31 mg/kg). The intensity of fluorescence is nonlinear. It approximately doubles when the ICG concentration is increased ten fold, that is, *C*_ICG_ = 65  *μ*M/L (3.1 mg/kg).

The fluorescent light proceeds through the layer of tissue, which again attenuates the irradiance by the factor of *T*_*t*_. The remaining irradiance is scattered from the surface of the skin. If a Lambertian surface is assumed, the radiant intensity emitted from the tissue, *I*_*t*_, can be obtained from the irradiance of the skin, *E*_*F*,*t*_, as follows: *I*_*t*_ = *E*_*F*,*t*_/*π* (Table 9; row 7). 

Before hitting the sensor, the radiant intensity, *I*_*t*_, is attenuated by the emission filter, *F*_*c*_. Approximately *T*_*F*_*c*__  = 30% of the energy of the ICG fluorescent spectrum goes through the filter. Therefore, the intensity of light *I*_*c*_ entering the camera optics is *I*_*c*_ = *I*_*t*_ *T*_*F*_*c*__. (Table 9; row 8). The performance of the optics is often expressed as the so called *f*-number. The radiance in the image plane, *E*_*b*_, is obtained from the radiant intensity using the *f*-number of the optics, as follows: *E*_*b*_ = *I*_*t*_/*f* ^2^ (Table 9; row 9). 

As we have seen, only a small fraction of the initial light intensity induces fluorescence which finally will reach the image plane. To compensate the low light intensity, the exposure time, *t* [s], must be relatively long, which increases the signal level, *S* = Φ_*s*_ *E*_*b*_ *t*, where, Φ_*s*_, is the quantum efficiency of the sensor. Long exposure time also increases the level of thermal noise, *N*_th_ [e/pixel], due to dark current, *i*_*d*_ [e/pixel/s]. The total thermal noise within the exposure interval, *N*_th_ = *i*_*d*_ *t*, dominates the total noise, *N*_*r*_ [e/pixel], when *t* > *N*_*r*_/*t*. Above this limit, increasing the exposure time increases the total noise level, *N*_tot_ = *N*_*r*_ + *N*_th_, approximately at the same rate as the signal level, and thus the signal to noise ratio, SNR = *S*/*N*_tot_, is not significantly improved any more. Therefore, the light entering the image plane should have sufficient intensity to keep the exposure time short enough. 

The optimal exposure time *t*_*o*_ can be determined, if the physical pixel area, *A*_*p*_, and the maximum number of electrons the pixel can hold, the quantum well depth, *D*_QW_, are known. The number of photons hitting the pixel is *E*_*b*_ *A*_*p*_/*E*_*p*_, where *E*_*p*_ = *ch*/*λ* is the energy of the photon, where *c* is the speed of light, *h* is Plank's constant, and *λ* is the wavelength of the photon. Therefore, the time which is needed to fill the quantum well is

(1) $$\begin{array}{c} t_{o}=\frac{D_{\text{QW}}E_{p}}{\Phi_{s}E_{b}A_{p}}. \end{array}$$

As an example, the calculation of the observed fluorescent intensity and the performance of the Hamamatsu ER-150 sensor is estimated in Table 9 (rows 10–14). Many parameters used in the calculation are only estimations, especially the tissue properties above blood layer, the haemoglobin concentration in blood, the concentration of the ICG and the quantum efficiency of ICG. Furthermore, the fact that the emission spectrum of ICG may depend on the spectrum of the illumination is neglected. Therefore, the absolute values given in the above calculations are not accurate. However, they provide insight into the losses in the imaging system. Eventually only about 35 ppm (parts per million) of the original radiation intensity remains in the image plane, and only about 25 percent of it will be detected. The summary of top five loss factors causing −48.7 dB attenuation of the total −50.5 dB attenuation in the example system is shown in Table 10. Remember that one full stop used in camera lenses is equivalent to 3 dB. Thus, the total attenuation corresponds to about 17 full stops, which means that we need to use large aperture, long exposure time, and strong illumination in order to get high quality, that is, high SNR ICG fluorescence images.

#### 3.2.4. Test and Background Light

 As NIR light is not visible to the human eye and fresh ICG-water solution is not always at hand, it is practical to have a test light to see if the camera system is working on the ICG fluorescence wavelengths. We have used an LED SFH485-P (Osram/Siemens, Berlin, Germany), having peak emission at 880 nm, as a test light to see if the camera is tuned to wavelengths ranging from about 800 nm to 900 nm. We have also constructed a simple light control for using this LED as a background light for ICG fluorescence imaging. The test light can also be used as tunable backlight, when we want to see landmarks not fluorescing themselves.

#### 3.2.5. Optics

**Figure 6:**
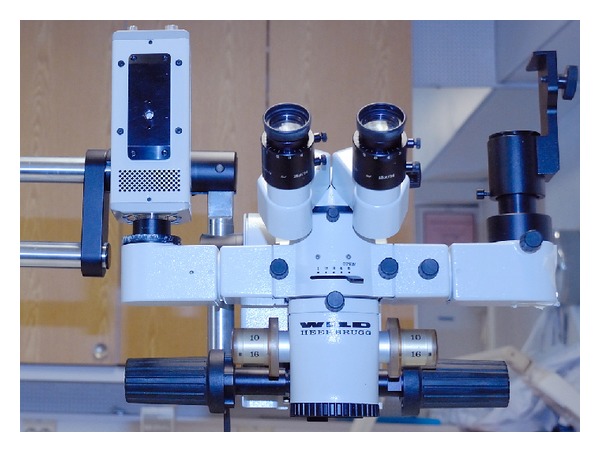
An old operational microscope used in our prototype ICG stereo video angiography system experiment. Hamamatsu NIR camera on the left camera arm.

Our example system was based on an old operational microscope originally not at all designed for NIR imaging (Wild, Figure 6). It has two oculars and a *C* mount for a camera for both eyes. This gives us an opportunity not only to record ICGA videos but also ICGA stereo videos. It has been shown that stereo videos are beneficial in the training of surgery students [114].

Excluding the microscope, the cost of our prototype components including two interference filters, two cameras, an LED light, and a PC with some software is about 3000 euros. Figure 1 shows a typical image taken by our prototype system. As can be seen the quality of the image is quite good, especially when we remember that the optics used (the microscope) is not designed for NIR imaging. The use of special NIR optics would considerably increase the cost of the system while probably only somewhat increasing the image quality.

#### 3.2.6. ICGA Test

After technical laboratory tests, our device was tested by recording ICGA of rat heart (Figure 4). Neither the use of ICG dye nor the modest optics not originally designed for NIR imaging does too much restrict resolution, when compared to imaging in visible wavelengths: arteries, the caliper of which are only a fraction of millimeter, can be clearly seen.

Using two cameras, stereo images and stereo video can be taken. The cameras can be attached to an operational microscope (Figure 6) or simply attached together when microscopy is not used. This gives literally new vision for ICGA, especially for educational and training purposes when complex scenes can be seen stereoscopically.

## 4. Surgical Applications

 Established medical applications of ICG are retinal angiography, liver clearance test, and cardiac output monitoring. ICG is fast removed from circulation by the liver into bile juice, which is applied in liver condition monitoring. It also gives the option to inject ICG several times during an operation if needed. Recent interest in ICG is based on new applications in surgery and especially in angiography related to intraoperative monitoring of blood circulation in vital organs, where intraoperative angiography is also economically motivated [115].

### 4.1. Intraoperational Angiography

As compared to other angiography methods (X-ray, CT, MRI, and PET), ICGA can be easily and economically used intraoperationally, when blood vessels are exposed allowing direct visual observation, for example, in neurosurgery, bypass coronary surgery, flap operations in reconstructive surgery, wound and trauma surgery, and laparoscopic surgery, where it is vital to check that blood circulation is recovered properly.

The imaging protocol is simple, and devices are relatively cheap. ICG is given as an injection (bolus) into systemic blood circulation and imaging is done during a period of few minutes after injection. Normally a new bolus can be given after about 15 minutes.

### 4.2. Neurosurgery

Neurosurgery is ideal for ICGA because operations are already done under a microscope (and camera), and because the blood veins located on the brain surface are mainly exposed and thus can be seen more or less directly by visual means. Milestones in neurosurgery include

- 2001: experiment with surgical microscope (OPMI) in neurosurgery [97];
- 2002: FDA approval for cerebral angiography research [102];
- 2003: ICGA was introduced for clinical neurosurgery [88];
- 2005: ICGA done with surgical microscope [116];
- Leica 2006: FDA approval of ICGA surgical microscope;
- Zeiss 2007: commercial surgical microscope with ICGA;
- Zeiss 2009: ICGA dynamics display software.

Earlier, ICG has been used in neurology, for example, for measurement of cerebral blood flow in newborn infants [117].

 Neurosurgical vascular operations are usually performed to exclude vascular malformations from the circulation or to provide revascularisation in case of compromised cerebral perfusion. Typical vascular anomalies to be treated surgically are cerebral aneurysms and intracranial or intraspinal arteriovenous malformations (AVMs) and fistulas. It is of utmost importance to be able to verify that the malformation in question has been completely obliterated and removed from the circulation, and just as critical is to ensure that physiological blood flow in associated and adjacent vessels remains uncompromised at the end of the procedure. In revascularisation, that is, bypass procedures, the patency of the vascular microanastomosis is likewise paramount to successful procedures. Incomplete obliteration of a rupture-prone aneurysm or AVM may result in a hemorrhage, and occlusion of a parent vessel or an anastomosis in an ischemic stroke; both of which may have catastrophic consequences for the patient. Postoperative angiography is useful in assessing the residual filling of the treated lesion, but in case of inadvertent vessel occlusion the result of postoperative imaging comes too late, and the ischemic brain or medullary lesion has already irreversibly occurred. Although it is possible to use intraoperative digital subtraction angiography (DSA) in the operating room, the setup takes a relatively long time, and thus DSA cannot be used routinely in every operation. Moreover, DSA is associated with a complication rate of up to 3%, and its resolution is insufficient to demonstrate the occlusion of small (<1 mm) perforating arteries, which, despite their small caliber, may supply blood flow to critical neural structures in, for example, basal ganglia and the brain stem. In neurosurgery all complex operations are performed under high magnification of a surgical microscope, which provides an excellent hardware platform for implementing new optical solutions and to mount various external devices, such as video cameras. 

ICG angiography was introduced to neurosurgery in 2003 [88] and has become a routine method for intraoperative evaluation of intracranial blood circulation. It has been used at the Department of Neurosurgery at Helsinki University Central Hospital since 2005 in approximately 300 operations every year. It provides real-time information about the patency of vessels of all sizes seen in the field of the surgical microscope. Its usefulness in intracranial aneurysm surgery has been recently assessed in several large patient series, in altogether 620 aneurysms [116, 118–121]. The uniform conclusion of all the reports was that the correlation between ICG angiography and postoperative angiography has been 90–95%, in terms of aneurysm remnants and vessel branch stenoses or occlusions. In addition, ICG angiography has the added advantage of demonstrating small perforating artery occlusion intraoperatively, enabling the immediate correction of aneurysm clip placement [118]. However, ICG angiography may be inadequate in cases of giant, complex, or deep-sited aneurysms [119]. Atherosclerotic calcifications also limit its reliability in demonstrating, for example, aneurysm neck remnants.

During microneurosurgical treatment of brain or spinal arteriovenous malformations and dural arteriovenous fistulas, the dynamic visualisation of different phases of the blood flow by ICG angiography is helpful in the identification and differentiation of feeding arteries, arterialised draining veins, and normal veins, as well as the fistulous sites, during intraoperative orientation within the surgical field [87, 122–124]. It should be noted, however, that especially brain AVMs often have complex 3D anatomy, and ICG angiography is only able to visualise vessels on the surface of the illuminated surgical field. Likewise, ICG angiography cannot, at present, be considered reliable in assessing possible residual AVMs, which still requires DSA either intra- or post-operatively. 

ICG angiography has also been evaluated and found reliable in assessing the patency of microanastomoses in neurosurgical extracranial-intracranial revascularisation bypass operations [125]. It was also helpful in identifying the target recipient artery of sufficient diameter (>1 mm) in extracranial-intracranial bypass procedures performed via very small (3 cm) craniotomies [126]. ICG-VA has also been demonstrated to be useful in evaluating the patency of extracranial vertebral artery after surgical transposition and in localizing vertebral artery within its periosteal sheath during surgery of cervical neurinomas [127]. Very recently, Haga et al. have used ICG-VA for assessment of carotid endarterectomy [128]. Commercially available semiquantitative dynamic ICG fluorescence analysis system has also been recently suggested to be able to demonstrate impaired regional perfusion in patients with cerebral ischemia [129].

The usefulness of ICG angiography in microneurosurgical vascular operations is increasingly acknowledged, as more applications are developed, and more experience is gained. However, there is still room for technical developments, for example, in form of rapid and reliable flow dynamics analyses and easily processable and repeatable video playback loops, since rapid ICG reinjections generally suffer from lower contrast due to residual ICG inside the vessels.

### 4.3. Coronary Surgery

In principle, coronary arteries are also ideal for ICGA because they are located, like brain arteries, on the organ they supply blood to. The major milestones of ICG in coronary bypass surgery include

- 2002: a pig model of coronary bypass angiography with ICG [130];
- 2004-2005: comparison of ICGA and ultrasound flow metering at University of Toronto;
- 2005: FDA approval for ICGA device SPY for coronary angiography;
- 2005–2009: GRIIP clinical trial (phase III) at Sunnybrook Health Sciences Centre.

Coronary artery bypass grafting (CABG) is the most frequent cardiac operation with annual rates of 400,000 procedures in the United States and 76,000 in Germany. During these operations verification of graft patency should be a key aspect, as immediate intraoperative graft failure occurs in up to 4% of grafts (8% of patients) [131]. At patient discharge the graft occlusion rate is 5–20% and up to 30% at one year after the operation [132]. Intuitively, eliminating intraoperative graft failure and technical failure should reduce cardiac mortality and morbidity in the short term and improve clinical outcome in the long term. Although conventional angiography remains the gold standard technique for assessing graft patency, it is rarely available in the operating room and consequently several other less invasive approaches have been advocated. The most commonly used intraoperative method is transit-time flowmetry (TTFM), which measures the mean flow of the bypass graft and calculates a pulsatility index of the flow pattern. TTFM is reliable and sensitive in detecting graft failure, but in several patients it might lead to unnecessary graft revision [132]. 

Near-infrared imaging (NIR) based on the intravascular ICG dosing has emerged as a novel method for graft patency assessment. Two main systems have been introduced. 

Firstly, an indirect method in which the myocardial tissue perfusion is assessed by imaging an area of interest around a coronary vessel. In this imaging method, peak fluorescence intensity and temporal slope of fluorescence intensity in the tissue are measured [130]. This imaging method has been shown to agree with the result of the fluorescent microsphere imaging, which is the golden standard [133]. 

Secondly, a direct imaging of the grafts by visualising the graft lumen by ICG angiography. In an early study by Rubens et al., 20 patients were studied by intraoperative ICG angiography, and one patient (5%) was identified as needing a graft revision [134]. Taggart et al. investigated 213 grafts with a revision rate of 4 grafts (1.9%) acknowledged by ICG angiography [135]. Reuthebuch and colleagues published a graft revision rate of 4 (3.7%) out of 107 patients [136]. Balacumaraswami et al. assessed the intraoperative graft patency of 533 conduits in 200 patients. Fluorescence imaging confirmed technical failure in 8 (1.5%) conduits in 8 (4%) patients, necessitating graft revision [137]. Takahashi et al. reported a study of intraoperative ICG angiography of 290 grafts, in which four grafts (1.9%) were visualised to need a graft revision [138]. In a paper by Desai et al., a total of 348 coronary bypass grafts were studied by ICG angiograms. In 4.2% of patients information from the ICG imaging led to graft revisions that would have otherwise gone unrecognised [139, 140]. 

Intraoperative graft occlusion in CABG is a consistent finding affecting up to 5% of grafts. This probably causes difficulties in both the short and the long term. Detection of technical problems in the most vital graft, the internal thoracic artery is of utmost importance. Among the available techniques for assessing graft patency, intraoperative ICG angiography seems to provide a sensitive method compared to the mostly used method of TTFM. In a recently published randomised trial, 156 patients were randomised to go through ICG angiography or TTFM during CABG to assess graft function intraoperatively. One year after the operation, 43 out of 312 grafts were occluded (13,8%), with no difference between the groups. Thus, ICG angiography seems to provide a novel technique in addition to the more acknowledged range of methods of intraoperative quality confirmation in coronary surgery [96]. 

### 4.4. Vascular Surgery

 In vascular surgery, ICG fluorescence imaging has been studied in intraoperative assessment of graft patency, diagnostics of peripheral arterial occlusive disease and Raynaud phenomenon (RP) as well as in predicting wound healing after major amputation and to evaluate splanchnic circulation. Also, the usefulness of ICG angiography in evaluating angiogenesis in small animal models and in detecting the vulnerability of atherosclerotic plaque has been tested. In one study ICG imaging was used in the treatment of varicose veins with sclerotherapy.

 In a preliminary report by Unno et al., 9 patients were recruited in an intraoperative angiography performed with PDE. At the end of the operation before wound closure, ICG was injected in a central intravenous line. ICG dye reached the leg artery about 30 seconds after the injection. In 8 out of 9 cases, ICG angiography showed good fluorescent signals as the ICG passed through the graft. In one case no fluorescent signal was detected and during revision a distal thrombosis was detected and repaired [141].

 Kang et al. have proposed a perfusion rate model based on ICG dynamics, which they later apply to human patients to diagnose peripheral arterial occlusive disease (PAOD) with VasView [90]. PAOD patients and control subjects with normal vasculature were evaluated for lower extremity tissue perfusion using ICG perfusion imaging. The perfusion rates of the lower extremities with severe PAOD were significantly lower than those of normal controls. Even in cases of mild PAOD, the perfusion rates were lower compared to the control, while the conventional methods failed to detect mild functional impairment. These results collectively indicated that ICG perfusion imaging is an effective tool for diagnosis of PAOD, when compared to the golden standard of ankle-brachial blood pressure ratio [101, 142].

 In a recent study, Kang et al. tested the use of combined analysis of multiple parameters, especially onset time and modified *T*_max⁡_, which means the time from onset of ICG fluorescence to *T*_max⁡_, to diagnose Raynaud phenomenon (RP). To validate the method, they performed a conventional thermographic analysis combined with cold challenge and rewarming along with ICG dynamic imaging and segmental analysis. A case-control analysis demonstrated that the segmental pattern of ICG dynamics in both hands was significantly different between normal and RP cases, suggesting the possibility of clinical application of this method for the reliable diagnosis of Raynaud phenomenon [143]. 

In patients with no possibility of revascularization, about half sustain amputation within one year. To maintain best possible mobility, amputation should be done as distally as possible. On the other hand, healing of the amputation wound should be assessed before the procedure to avoid wound healing problems, infections, and reamputations. Zimmermann et al. evaluated the use of ICG fluorescence angiography at an early postoperative time point to predict the tissue necrosis at the level of amputation. The perfusion of amputation stumps was measured with the IC-View-System. In total 10 patients with critical limb ischemia and ischemic tissue loss were investigated within 72 hours after major amputation (above knee and below knee) with indocyanine green (ICG) fluorescence [144]. 

Strategies for neovascularization of ischemic cardiac or lower extremity tissue has been under intensive research recently. For example, gene technology has been studied to achieve therapeutic angiogenesis for peripheral arterial disease. One major problem in this investigation has been visualization and quantification of collateral growth in small animal models. The current gold standard of minimal invasive determination of blood perfusion within the hind limb of mice is the laser Doppler perfusion imaging (LDPI). However, it does not penetrate the entire limb and, thus, measures relative superficial perfusion rather than collaterals in muscle layer. Wuestenfeld et al. evaluated the applicability of the ICG angiography for the determination of hind limb perfusion in mice and compared it to LDPI. The authors suggest that ICGA is a potent tool for the quantification of collateral flow in small animal models and that LDPI shows unreliable high perfusion in the operated foot after one week indicating that it measures perfusion in the superficial skin rather than entire hind limb [145].

 Lipid rich vulnerable plaques are the main cause of acute vessel occlusion in atherosclerosis. It has been recognised that ICG is a lipophilic molecule that accumulates at sites of lipid and inflammation. In animal models, it has been shown that ICG accumulates in lipid in aortic plaques and helps localise the atheromas. Furthermore, in human carotid artery specimens it has been demonstrated that ICG colocalised with lipid-rich atheroma and macrophages. Together these results suggest that ICG may be useful as a imaging agent specifically for lipid-rich and inflammatory atherosclerotic vessel lesions [146].

 ICG fluorescence imaging has also been used to measure splanchnic blood flow. Leppikangas et al. studied the effects of levosimendan on systemic and splanchnic circulation during and after abdominal aortic surgery in a double-blinded randomized study, in which 10 patients received levosimendan and 10 patients placebo. The total splanchnic blood flow was estimated by measuring the indocyanine green plasma disappearance rate (ICG-PDR) transcutaneously. Each patient was connected to an ICG finger clip, which was connected to a liver function monitor (LiMon). A 0.25 mg/kg dose of ICG was injected through a central venous line of the pulmonary artery catheter at baseline, before and during aortic clamping, and postoperatively. Levosimendan did not have a significant effect on total splanchnic perfusion in patients undergoing an elective aortic aneurysm operation [147].

Foam sclerotherapy is a widely used treatment for varicose veins. The spreading of the sclerosant is usually visualized by ultrasound. Kikuchi et al. reported the development of visualized sclerotherapy procedure using PDE. Camera images were digitized for real-time display and reviewed. Operating lights were turned off during imaging. ICG was mixed with polidocanol and air. In total, 35 patients were treated and studied. In all patients, sclerosant spreading was seen as excellent, and no side effects from ICG were observed [148].

### 4.5. Oncology and Sentinel Lymph Node Harvesting

 The pioneering work of Chen et al. using a rat model shows that ICG injected in cancer tumor can be used in laser assisted photothermal- and photoimmuno-therapies [149, 150]. The first clinical trials of this kind of therapy have recently done successfully by the same group [151].

Lymph nodes are the initial site for metastases for most cancers. According to surgical principles, all cancer tissue within the primary tumour and metastatic lymph nodes should be removed during the surgical operation in order to achieve a complete and potentially curative resection. The sentinel node is the lymph node that receives the first lymph flow from a malignant tumour, and universally it is the first station, where a potential dissemination of malignant disease can be identified [152]. A real problem in cancer surgery is that the lymph nodes are difficult to harvest during operation. Currently radioactive technetium-99 m isotope labeling is used to detect lymph nodes. This may be replaced by ICG NIR imaging. This attractive method to facilitate the visualisation of lymphatic vessels, sentinel nodes, and metastatic lymph nodes has been introduced by Lim and Soter [153]. Here, ICG is injected under the skin from where it flows via lymph circulation to lymph nodes revealing them when lit with excitation light [92, 98, 154]. More recently, Kim et al. have used a dual-modality lymph node mapping to detect sentinel lymph nodes in rats, combining photoacoustic and fluorescence imaging [155]. Ito and colleagues utilised sentinel node navigation based on ICG in patients who were diagnosed to have lung cancer [156]. They concluded sentinel node navigation using ICG in lung cancer to be feasible, but some modifications will be necessary before the method can be clinically applied. Following the clinical approval of ICG for cutaneous Kaposi's sarcoma and cutaneous metastases of a rectal carcinoma they have been treated successfully [157–159]. Crane et al. have very recently used ICG in transcutaneous SLN detection in vulvar cancer patients [160] and Inoue et al. for identification of lymphatic pathway involved in the spreading of prostate cancer [161]. 

Albumin affects ICG fluorescence efficiency. Therefore in some studies albumin, usually human serum albumin (HSA), has been mixed with ICG in order to increase the fluorescence efficiency and thus sensitivity of ICG in SLN detection. However, a very recent randomised, double-blind comparison of ICG with and without HSA seems to indicate that there is no increase in sensitivity at least in the case of breast cancer [162]. 

We believe that the possibility to identify lymphatic vessels and appropriate lymph nodes in the operating room during surgery would yield marked benefits in terms of completeness of surgical resection and perioperative evaluation of potential dissemination of a malignant and deadly disease.

#### 4.5.1. Lymphography

 The lymphatic system is vital for many physiological processes, including immune reactions, and the maintenance of body fluid and chemical balances. The lack of noninvasive methods to monitor lymphatic pumping dynamics has been perhaps the most important reason for keeping the role of lymphatics modest in the clinical setting. 

Unno et al. have recently shown how ICGI can be used in a minimally invasive method of monitoring human lymphatic pumping with a commercially available device and a custom-made transparent sphygmomanometer [163].

### 4.6. Liver Surgery and Laparoscopy

 ICG has been used for many years as a test for hepatic function and to measure hepatic blood flow in humans and different animal species [164]. In these tests, ICG clearance has mainly been assessed by its blood clearance curve [165, 166]. There are a limited number of studies evaluating the hepatic blood flow and liver function by direct ICG clearance using NIRS in healthy rabbits and rabbits with surgically reduced hepatic blood flow or experimentally induced liver cirrhosis [167]. The studies showed that measurement of ICG clearance by NIRS is promising for the assessment of liver dysfunction and may have applications in hepatic surgery and transplantation. Furthermore, the technique reflects the reduced liver blood flow and perfusion in liver cirrhosis more accurately than the previously used peripheral blood ICG clearance. The ICG excretion determined by NIRS correlated with the degree of parenchymal liver dysfunction [167]. A recent study in humans revealed that the measurement of hepatic ICG uptake by NIRS could become a valuable tool for assessing the indication for venous reconstruction in living donor liver transplantation and/or split donor liver transplantation. All studies mentioned show the potentials of NIRS-based determination of ICG clearance for the assessment of parenchymal liver function and perfusion. However, the absorption intensity of the liver after ICG injection by NIRS was determined in all studies by attaching NIRS sensors to the liver during laparotomy [168]. Injection of ICG via portal vein and subsequent imaging can be used to intraoperationally visualise liver segments and subsegments [169].

Ishizawa et al. have used ICG in a routine liver test preoperationally, after which a prototype ICG fluorescence imager was used to detect hepatocellular carcinoma intraoperationally during a laparoscopic hepatectomy [93].

 In 2009, two clinical studies revealed that real-time ICG-fluorescent imaging enabled the highly sensitive identification of small, grossly unidentifiable liver cancers. This led to an enhanced accuracy of operative staging and liver resection. Currently, indocyanine green fluorescence imaging navigation is considered to be a promising tool for clinical exploration for hepatocellular carcinoma and for routine intraoperative imaging during hepatic resection [170, 171].

 Sentinel lymph node detection has been one main application of ICG in laparoscopic studies including early gastric cancer treatment and gastrectomy [172–177]. Harada et al. compared conventional and ICG-based laparoscopic sentinel node mapping for colorectal cancer and conclude that the latter is superior to the former [178]. Miyashiro et al. have recently applied a prototype ICG laparoscopic system [179] to detect sentinel nodes in gastric cancer surgery [180]. Jeong et al. have developed a new NOTES procedure for laparoscopic sentinel lymph node dissection of the stomach with ICG marking using a pig model [181]. 

Note, that CO_2_ pneumoperitoneum used in laparoscopic operations, which decreases liver blood flow, also increases ICG half-life [182].

**Laparoscopic Cholecystectomy**

While ICG is rapidly excreted via the bile duct, it is most natural to apply ICG intraoperationally to aid bile examination and operations [183]. Indeed, several groups have recently showed how ICG can be used to aid both open and laparoscopic cholecystectomy [184–189]. An earlier work by Ikeda et al. uses ICG for monitoring primary sclerosing cholangitis [190].

### 4.7. Reconstructive Microsurgery

 For the past 20 years, the use of different fasciocutaneous perforator flaps has become popular in the field of reconstructive plastic surgery. Flaps that are over 15 × 30 cm in size can be raised on a single perforating artery and its concomitant vein. These flaps have been used to reconstruct a wide range of different tissue defects. Even a partial loss of the flap can lead to the total failure of the reconstruction. In perforator flaps, the perfusion of the most distal parts of the flaps is often problematic. Recently, several reports have shown the feasibility of ICG angiography in the intraoperative assessment of flap viability [191–199]. ICG angiography has also been used to assess the patency of the microvascular anastomosis intraoperatively, and the intrinsic transit time of the flap circulation with promising results [200–202]. In the preoperative planning of perforator flap reconstructions, ICG angiography has been used to detect suitable perforator vessels, which facilitates the design of the flap [203]. After free tissue transfer surgery, the flap circulation has to be carefully followed. Although other methods exist for continuous flap monitoring, ICG angiography may be helpful in the early postoperative phase for the detection of anastomotic thrombosis, when flap survival is in doubt [144, 202, 204–207]. In large axial or random pattern flaps, ICG angiography can be used intraoperatively for deciding the need for a delay procedure to ensure flap survival [205, 208–210]. In breast reconstructions, transverse abdominal flaps can be raised on superficial vessels alone (SIEA-flap) or more commonly using the deep inferior epigastric perforator flap (DIEP or TRAM flaps). The choice is often made intraoperatively, and ICG angiography is found to be a beneficial tool in the decision making [211–216]. There has also been interest in using ICG angiography in evaluation of tissue viability, especially in traumatic degloving wounds and burns. This evidence is sparse, though, and further research is needed [191, 217–219].

 Intraoperative assessment of flood flow is important for successful transplantations. Hoffmann et al. have used laser-assisted ICGA (IC-VIEW) for successful intraoperative assessment of kidney allograft perfusion in 10 consecutive *de novo* renal transplantations [220]. Sawada et al. have recently used ICGA by PDE for intraoperative assessment of renal vasculature after revascularisation of a transplanted kidney [221]. Mizuno and Isaji have used ICG-injected intrabiliary for donor liver bile duct imaging [222].

### 4.8. Other Clinical Applications

 In addition to the above surgical applications, the clinical application of ICG include such topics as brain imaging and hemodynamics [223, 224], rheumatoid arthritis [225–227], burns and other trauma [228], and muscle perfusion [229].

Due to the absorption at wavelengths under 800 nm, ICG can be used with a suitable intense light source, typically a laser, as an ingredient of a tissue solder like albumin, which readily bounds to ICG [230–238]. ICG-based tissue soldering could be one method for making surgery more automatic as laser-based ophthalmic surgery is already today.

#### 4.8.1. Photodynamic and Photothermal Therapy

 When an ICG molecule is excited, it can further transfer energy to other molecules. When exciting oxygen, ICG turns out to be a photodynamic therapy agent. In principle, for example, after having been used to reveal lymph nodes a strong illumination with NIR light could be used to destroy metastatic nodes. ICG binds easily to tissue even at high concentrations, and the visual change in colour from green to orange is manifested by the wavelength shift in reflectance peak. ICG has been used *in vitro* laser-assisted fat cell destruction, which might give a new optics-based procedure for cosmetic surgery [239].

Similarly, ICGA can be used as a light-activated antibacterial agent (LAAA), for example, in wound healing [234], or treating chronic rhinosinusitis [240] with near-infrared laser illumination (NILI). It was shown recently that the photodynamic effect can be used for acne treatment [241–243]. Nevertheless, many problems have to be solved in order to design optimal technology for acne treatment without side effects.

Through intense light (laser) irradiation a number of new effects can be provided, which lead to more effective bacteria killing and controllable cell destruction and/or inhibition of excessive synthesis of sebum in sebocytes, like the localised photodynamic effect based on the appropriate concentration of the suitable exogenous dye incorporated into hair follicle or any other skin appendages. The indocyanine green is one of the prospective exogenous dyes for soft photodynamic treatment (PDT).

### 4.9. Dyeing

 Finally, as ICG is a dye it can be naturally used for tattooing, labeling, and similar tasks [244–249]. A relatively early work demonstrates how ICG can be used to stain caries lesions for the further removal of lesion by a laser by the help of the high light absorption of ICG at the excitation wavelength [250]. Recently, Kitai et al. have used ICG for monitoring of perineal wound contamination in abdominoperineal resection [251]. 

Because hepatocytes handle ICG in the liver, ICG can be used also to monitor differentiation of mouse embryonic stem cells into hepatocytes [252, 253].

## 5. Conclusions

In this work, we have reviewed well over 200 papers describing the development and use of the fluorescing contrast agent indocyanine green in clinical, mainly surgical, applications. Many interesting works had to be omitted simply due to space limitations. However, it is hoped that we have succeeded in collecting most of the key publications for giving an overview of the indocyanine green fluorescence technology and its most important emerging clinical application.

Many new clinical applications of ICG and ICG angiography are just emerging and more are definitely expected to appear in the near future; thus, it is obvious that much more research is still needed in order to fully realise all the potential of this relatively simple optical technology. The obvious fields of further engineering research include

- image processing of ICG and ICGA information, also in real-time (video and stereo) [143, 239, 254, 255],
- capillary circulation monitoring and perfusion dynamics imaging [256–262],
- combining ICG and other imaging modalities like visual, CT, MRI, and PET [263–266],
- combining ICG and ICGA with dermal imaging methods [110],
- deeper imaging (optical tomography) [261, 267],
- optical imaging device development (laparoscopy) and optimisation, hands-free [4, 268, 269],
- development of new derivatives of ICG for more specific imaging modes,
- increasing the quantum efficiency of ICG by, for example, metallic nanoparticles [270, 271],
- micro- and nano-encapsulation of ICG for nonangiography applications [76, 272],
- extraction of spectral information and chemometry (multispectral imaging) [273], and
- integration of ICG imaging to robotic-assisted surgery [274].

In a clinical setting, ICG is a new and unique method in imaging of the lymphatic circulatory system and thus offers both challenges and the potential for totally new clinical applications [27].

## Figures and Tables

**Table 1:** The number of ICG-related publications: queries from databases PubMed, ISI, SPIE, and IEEE (26.7.2011).

*Keyword*	PubMed	ISI	SPIE	IEEE
“Indocyanine” (ICG)	6069	5159	301	57
ICG and “surgery”	2160	1059	25	4
ICG and “liver”	2031	1164	18	5
ICG and “retina”	1176	406	7	11
ICG and “cancer”	816	372	49	14
ICG and “tomography”	748	594	29	10
ICG and “imaging”	697	828	130	43
ICG and “heart”	483	174	3	0
ICG and “wound”	190	53	24	1
ICG and “lymph”	128	115	11	1
ICG and “brain”	127	119	16	0
ICG and “breast”	105	189	35	10
ICG and “laparoscopy”	47	26	0	0

**Table 2:** From PubMed (2.11.2009) “Indocyanine”: (years 2007–2010: 17.8.2011).

Recent		Early	
Year	Number	Year	Number
2010	397	1970	38
2009	369	1969	35
2008	295	1968	29
2007	275	1967	37
2006	274	1966	25
2005	277	1965	20
2004	295	1964	16
2003	295	1963	8
2002	240	1962	5
2001	219	1961	2
2000	224	1960	10
1999	195	1959	0
—	—	1958	1
1989	96	1957	0
—	—	1956	0
1979	69	1955	0
—	—		

**Table 3:** ICG cytotoxicity studies.

Cell	Type	Ref.	Comment
ARPE19	*In vitro*	[54]	ICG 1 mg/mL; not toxic
RPE	*In vitro*	[55]	IfCG; no damage
Glial cell	Ditto	Ditto	ditto; some damage
RPE	Rabbit	[56]	ICG; no damage
RPE	Ditto	Ditto	IfCG; no damage
RPE	Gene expr.	[57]	Cell cycle arrest and apoptosis
Ditto	Ditto	Ditto	0.25 mg/mL recommended
Müller	*In vitro*	[41]	Fluorescent lamp illumination:
Ditto	Ditto	Ditto	induces cytotoxicity
Intravenous	Rat	[58]	IRDye 800CW; no toxicity obs.
Intradermal	Ditto	Ditto	Ditto; ditto
Spin. root ax.	Rat	[59]	Neurotoxicity observed
RPE	Human cult.	[60]	Growth inhibition and damage
RPE	Human cult.	[61]	ICG interactions with RPE

**Table 4:** ICGI instruments. KAIST: Korea Advanced Institute of Science and Technology.

Manufacturer	Device	Ref.	Comment
Carl Zeiss	Pentero IR-800	[87]	Surgical micr.
Carl Zeiss	Pentero?	[88]	Neurosurg. micr.
Cri, Inc.	Maestro	[71]	Small animal
Eastman Kodak	Imaging Station FX	[89]	Small animal
Eastman Kodak	Ditto 4000 MM	[90]	Small animal
Florida Int. U.	Prototype *FIU*_1_	[91]	Breast imager
Hamamatsu	PDE	[92]	
Hamamatsu	Prototype (*H*_1_)	[93]	Laparoscopic
KAIST	Prototype *AA*_1_	[94]	Small animal
Mizuho Ikakogyo	HyperEye	[95]	surgery
Novadaq Tech.	SPY	[96]	
Osaka Med. Coll.	Prototype *OMC*_1_	[97]	Neurosurg. micr.
Pulsion	IC-View	[98]	
Topcon	TRC-50IX	[99]	Ophthalmoscope
U. Clinic Munich	Prototype *UCM*_1_	[67]	Endoscope
U. Kent	Prototype *UK*_1_	[100]	OCT ophthalm.
Vieworks Corp.	VasView	[101]	Human leg im.
Wetzlar	Leica OH3 FL800	[102]	Surgical micr.

**Table 5:** ICGI instrument properties. *: Hitachi, *λ*_*e*_ [nm] emission wavelength (min), *λ*_*c*_ [nm] camera wavelength (min), and cCCD cooled CCD.

Device	Light	*λ*_*e*_	Camera	*λ*_*c*_	Ref.
*AA*_1_	LED	740	cCCD	820	[94]
FX	Halogen	755	cCCD	830	[89]
*H*_1_	Xenon	?	CCD	810	[93]
IC-View	LED?	780	CCD?	835	[98]
Maestro	?	710	?	800	[71]
*OM* *C*_1_	Halogen	760	KP-160*	820	[97]
Pentero?	Laser	780	?	835	[88]
PDE	LED	760	CCD	820	[103]
VasView	LED	760	CCD	830	[101]
*UK*_1_	SLD	793	?	807	[100]

**Table 6:** Light source properties.

Property	Halogen	LED	Diode laser
Wavelengths	Visual-NIR	Rather narrow	Monochromatic
Price	Cheap	Cheap	Relatively expensive
Maintenance	Some	Not much	Some
Power	High	Rather high	High (pulses)
Pulses	Mechanically	Electronically	Electronically
Speed	Slow	Quite fast	Slow-very fast
Stability	Poor	Good	Good-very good
Special	Visual imaging	Small size	Extreme performance
Benefits	Cheap	Easy to control	No filtering needed
Drawbacks	High power loss	Filter needed	Speckle pattern
	Filter needed	New tech.	White light needed

**Table 7:** Some commercial NIR camera sensors.

Sensor	Technology	Resolution	Application	Manufacturer
MT9P031	CMOS	5 Mpix	Consumer	Aptina
MT9V032	CMOS	0.36 Mpix	Surveillance	Aptina
KAI-11002	CCD	10 Mpix	Consumer	Kodak
Neo	sCMOS	5.5 Mpix	Scientific	Andor
iXon3	EM-CCD	1 Mpix	Scientific	Andor
FL-280	sCMOS	2.8 Mpix	Medical	Hamamatsu
ER-150	CCD	1.3 Mpix	Medical	Hamamatsu

**Table 8:** The most important pixel parameters of the above NIR camera sensors.

Sensor	ADC	Read	Dark	Pixel	Well
	resolution	noise	current	size	depth
	(bit)	(*e*)	(*e*/pix/s)	*μ*m^2^	·1000*e*
MT9P031	12	2.6	25	4.8	8.5
MT9V032	10	—	—	36	—
KAI-11002	16	17	—	9	60
Neo	16	1	10	6.5	25
iXon3	—	<1	—	13	80
FL280	12	3	—	13	18
ER-150	12	10	—	41	15

**Table 9:** An example of the light attenuation in an ICG imaging system.

Row	Component	Attenuation	Remaining intensity
1	LED 780-66-60	0.2	*I*_0_ = 1 W/sr
2	Fs, ET775_50x	0.8	*I*_*s*_ = 0.8 W/sr
3	Tissue irradiance		*E*_*t*_ = 80 W/m^2^
4	Tissue, 1 mm	0.45	*E*_*e*_ = 36 W/m^2^
5	Fluorescence	0.0027	*E*_*f*_ = 0.10 W/m^2^
6	Tissue, 1 mm	0.45	45 mW/m^2^
7	Lambertian S.	0.32	*I*_*t*_ = 14 mW/sr
8	Fc, ET845_55 m	0.3	*I*_*c*_ = 4.3 mW/sr
9	Irradiance, f/1.1	0.82	*E*_*b*_ = 3.5 mW/m^2^

Response of the Hamamatsu ER-150 low light			

10	Power per pixel		*P*_*p*_ = 0.15 pW
11	Photons per pixel		*N*_*p*_ = 6.1 · 10^5^ photons/s
12	Detected photons	0.25	*N*_*e*_ = 1.5 · 10^5^ electrons/s
13	Optimal exposure		*t* = 98 ms
14	Signal to noise		SNR = 63 dB

**Table 10:** The loss factors and corresponding attenuations [dB] of the top five loss factors in ICGA imaging.

Loss factor	Loss	dB
The fluorescence of ICG in blood	0.0027	−25.6
Losses in the tissue above the blood vessel	0.2	−6.9
Quantum efficiency of sensor Φ_*s*_	0.25	−6.0
Transmittance of the emission filter	0.3	−5.2
Diffusion losses in the lambertian surface	0.32	−5.0

Subtotal	13.4 · 10^−6^	−48.7

Other factors together	0.62	−1.7

Total	8.8 · 10^−6^	−50.5

## Abbreviations

3D: Three-dimensional
ALT: Anterolateral thigh flap
ASA: American Society of Anesthesiology Patient Classification Status
AVM: Arteriovenous malformation
BP: Blood pressure
BSA: Bovine serum albumin
BSS: Balanced salt solution
CABG: Coronary artery bypass grafting
CCD: Charge coupled device (camera)
CEA: Carotid end arterectomy
DSA: Digital subtraction angiography
EPR: Enhanced permeability and retention
FDA: (US) Food and drug administration
FLIM: Fluorescence Life-time imaging microscopy
FOV: Field of view FT fluorescence tomography
HDL: High-density lipoprotein
HAS: Human serum albumin
HUCH: Helsinki University Central Hospital
ICA: Independent component analysis
ICCD: Image-intensified CCD
ICG: Indocyanine green
ICG-PDR: ICG plasma disappearance rate
ICG-VA: ICG video angiography
ICGA: ICG angiography
ICGI: ICG imaging
ICG-PDR: ICG plasma disappearance rate
ICU: Intensive care unit
IfCG: Infracyanine green
IREE: Infrared-ray electronic endoscopy
ISPI: In situ photoImmunotherapy
LAAA: Light-activated antimicrobial agents
LDL: Low-density lipoprotein
LDPI: Laser Doppler perfusion imaging
LED: Light-emitting diode
LIFE: Laparoscopic intragastric full-thickness excision
MICAD: Molecular Imaging and Contrast Agent Database
MRI: Magnetic resonance imaging
NILI: Near-infrared laser illumination
NIR: Near-infra red
NIRF: NIR fluorescence
NIRS: NIR spectroscopy
NOTES: Natural orifice translumenal endoscopic surgery
OCT: Optical coherence tomography
PAD: Peripheral arterial disease
PAOD: Peripheral arterial occlusive disease
PASP: Sodium polyaspartate
PBS: Phosphate-buffered saline
PDT: Photodynamic therapy
PPG: Photoplethysmography
PPT: Photothermal therapy
PV: Peripheral vasculature
PVD: PV disease
QD: Quantum dot
RBC: Red blood cell
RGB: Red green blue
RP: Raynaud phenomenon
RPE: Retinal pigment epithelium
SLD: Superluminescent diode
SLN: Sentinel lymph node
SLNB: Sentinel lymph node biopsy
SNNS: Sentinel node navigation surgery
SNR: Signal-to-noise ratio
TTFM: Transit-time flowmetry.
